# *Bacopa monniera* Attenuates Scopolamine-Induced Impairment of Spatial Memory in Mice

**DOI:** 10.1093/ecam/neq038

**Published:** 2011-02-13

**Authors:** Manish Kumar Saraf, Sudesh Prabhakar, Krishan Lal Khanduja, Akshay Anand

**Affiliations:** ^1^Department of Neurology, Post Graduate Institute of Medical Education and Research, Sector-12, Chandigarh 160012, India; ^2^Internal Medicine, University of Texas Medical Branch, Galveston, TX 77555, USA; ^3^Department of Biophysics, Post Graduate Institute of Medical Education and Research, Chandigarh, India

## Abstract

Scopolamine, an anticholinergic, is an attractive amnesic agent for discerning the action of candidate antiamnesic drugs. *Bacopa monniera* Linn (Syn. Brahmi) is one such antiamnesic agent that is frequently used in the ancient Indian medical system. We have earlier reported the reversal of diazepam-induced amnesia with *B. monniera.* In this study we wanted to test if scopolamine-induced impairment of spatial memory can also be ameliorated by *B. monniera* using water maze mouse model. The objective of study was to study the effect of *B. monniera* on scopolamine-induced amnesia. We employed Morris water maze scale to test the amnesic effect of scopolamine and its reversal by *B. monniera.* Rotarod test was conducted to screen muscle coordination activity of mice. Scopolamine significantly impaired the acquisition and retrieval of memory producing both anterograde and retrograde amnesia. *Bacopa monniera* extract was able to reverse both anterograde and retrograde amnesia. We propose that *B. monniera's* effects on cholinergic system may be helpful for developing alternative therapeutic approaches for the treatment of Alzheimer's disease.

## 1. Introduction

The frequent causes of dementia include Alzheimer's disease (50%) [1]. Cognition deficits produced by cholinergic antagonism mimic the cognitive symptomology of Alzheimer's disease [2]. Scopolamine, a muscarinic receptor antagonist, is reported to impair Long term potentiation (LTP) [3], and frequently used as amnesic agent for evaluation of antiamnesic effect of new drugs. Considering the adverse effects of synthetic drugs [4, 5], there is search for natural remedies which are safe and effective. The World Health Organization (WHO) estimates that 80% of the world's population presently uses herbal medicine for some aspects of primary health care [6]. Therefore, natural products may provide a new source of beneficial neuropsychotropic drugs [5] provided they are adequately tested and their mechanisms are properly deciphered. Galantamine, a cholinesterase inhibitor and isolated from *Galanthus nivalis* and *Lycoris radiate* [7, 8], is useful for treatment of mild to moderate Alzheimer's disease [9, 10]. *Bacopa monniera* and *Centella asiatica* are the main constituents of Indian Ayurvedic herbal medicine which is known as *Medhya rasayana. Centella asiatica* is also reported to improve memory and promote the neuronal dendritic growth in hippocampus [11, 12]. The medicinal efficacy of *B. monniera* is also extensively reported in Indian traditional literature such as *Athar-Ved, Carak Samhita* and *Susrutu Samhita.* It has been extensively used for treatment of various neurological and neuropsychiatric diseases. *Bacopa monniera* extract has been reported to improve the memory in mice [13]. It contains bacosides A–F and nicotine as an active constituent [14]. It improves the performance of rats in various learning situations such as shock-motivated brightness-discrimination reaction, an active conditioned flight reaction and the continuous avoidance response [15]. Moreover, *B. monniera* also provides protection from phenytoin (an antiepileptic drug)-induced cognition deficit [16]. Bacoside A is the prominent constituent of *B. monniera* extract. It has been reported to alleviate the amnesic effects of scopolamine, neurotoxin and immobilization stress [17, 18]. *Bacopa monniera* improves the cognitive deficit possibly by exhibiting free radical scavenging and antilipid peroxidative effects [19]. There is evidence that the mechanism of action of *B. monneira* could be attributed to a combination of cholinergic modulation [20–24] and antioxidant effects [25–29]. Standardized extract of *B. monniera* was shown to improve the logical memory, paired associated learning and mental control in patients suffering from age-associated memory impairment (AAMI) [30]. Calabrese et al. [31] also demonstrated that *B. monniera* is a safe drug of choice to enhance the cognitive performance in elderly person. Chronic treatment of *B. monniera* also improves the cognitive function in adult [22] and children [32, 33], suggesting the usefulness of *B. monniera* in young community. In order to evolve better candidates for cholinergic receptors with minimal side effects [34], we examined the antiamnesic effects of *B. monniera* on scopolamine-induced amnesia.

## 2. Methods

### 2.1. Animals

All experiments were performed in accordance with the guidelines of Institute animal ethical committee and European Communities Council Directive (86/609/EEC). Adequate measures were taken to minimize pain or discomfort with animal experimental procedures. Swiss albino mice (male, age 3–5 months, weight 25–35 g) were housed four per cage with *ad libitum* access to food and water under controlled laboratory conditions. Experiments were conducted between 9:00 and 18:00 h in a semi soundproof laboratory. Healthy mice were screened on the basis of the swimming ability and normal behavior. Rotarod test in mice “before and after administration of scopolamine" was used to validate muscle coordination activity of mice. Mice, showing the normal fall time of 4 min from rotating rod at 15 rpm speed were selected for memory evaluation (data not shown).

### 2.2. Drugs and Chemicals

All the drugs solutions were prepared before use. The standardized extract of *B. monniera* (Lumen marketing company, Chennai), containing 55.35% bacosides, was suspended in 5% Tween 80, while scopolamine (Sigma-Aldrich, New Delhi) was dissolved in normal saline.

### 2.3. Morris Water Maze

Morris water maze [35] was used to assess learning and memory in experimental mice. There are several advantages of Morris water maze over other models of learning and memory [36] including absence of motivational stimuli such as food and water deprivation, electrical stimulations and buzzer sounds [37]. We followed the methodology of Morris water maze described in our earlier study. Briefly, it consists of a circular water tank, filled with opaque water, and one centimeter submerged platform. During acquisition trial escape latency time (ELT), time measure to locate the hidden platform, was noted as an index of acquisition. Each animal was subjected to the four acquisition trials per day for 6 consecutive days. The time spent by the animal, searching for the missing platform in target quadrant Q2 with respect to other quadrant (Q1, Q3 and Q4) on 7th day was noted as an index of retrieval. Rotarod test was performed to screen the muscle coordination activity of mice before subjecting them to water maze evaluation. Mice, showing abnormal swimming pattern in water maze, coupled with low muscle coordination activity in rotarod test were excluded from study. For studying the effect of drug on acquisition, the drug solution was administered before acquisition trial for 6 days. The diluent was administered before retrieval trial on 7th day. In order to test the effect of drug on retrieval of memory, the drug solution was administered before retrieval trial on 7th day. The study was divided in eleven groups. The number of mice in each group was 7. The details of drug treatment are described in Table 1. 

### 2.4. Data Analysis

All behavioral results were expressed as mean + standard error of mean (SEM). We have analyzed the behavioral results of Morris water maze using analysis of variance (ANOVA) followed by post hoc “Dunnet's" test or least significance difference (LSD) test. “a" indicates significance at *P* < .05 of particular day's ELT (i.e., ELT of Days 2–6) versus ELT on Day 1. We compared the ELT of treated group with control's ELT for each time point (i.e., Days 1–6). “b" indicates *P* < .05 versus ELT of control group for the same day. We also compared the ELT of treated group with ELT of 5% Tween 80 for each time point. “c" indicates *P* < .05 versus ELT of 5% Tween 80 group for the same day. The ELT of *B. monniera* treated group were compared with scopolamine's ELT for each time point. “d" indicates significance of ELT versus same day's ELT of scopolamine (0.5 mg kg^−1^ oral) group. “e" indicates significance of ELT versus same day's ELT of scopolamine (0.1 mg kg^−1^ oral) group. Retrieval test data were analyzed by ANOVA followed by least significance difference (LSD) test. “f" indicates *P* < .05 versus time spent in other quadrants, that is, Q1, Q3 and Q4. “g" indicates significance at *P* < .05 versus control group's time spent in target quadrant (Q2). “h" indicates significance at *P* < .05 versus 5% Tween 80 group's time spent in target quadrant (Q2). “i" indicates significance at *P* < .05 versus scopolamine group's time spent in target quadrant (Q2).

## 3. Results

### 3.1. Scopolamine Impairs Acquisition and Retrieval of Memory

**Figure 1:**
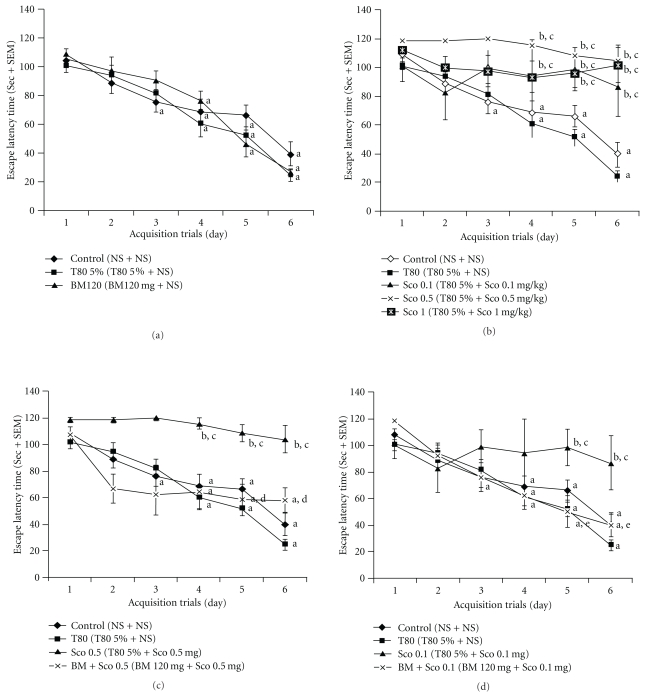
Effect of *B. monniera* on scopolamine-induced anterograde amnesia. In acquisition trials, each value represents mean ± SEM. Control group shows significant gradual reduction in escape latency time with acquisition days as compared with Day 1 ELT. “a" indicates significance at *P* < .05 of particular day's ELT (i.e., ELT of Days 2–6) versus ELT on Day 1, here data were analyzed using one-way ANOVA followed by Dunnett's test. (a) 5% Tween 80 does not affect normal acquisition as compared to control group. Similarly, *B. monniera* (120 mg kg^−1^ oral) does not affect normal acquisition as compared to control group and Tween 80 group. (b) Scopolamine significantly impaired the gradual reduction in ELT with acquisition days at dose of 1, 0.5 and 0.1 mg kg^−1^ as compared to control group and Tween 80 group. We compared the ELT of treated group with control's ELT or 5% Tween 80 ELT in each time point (i.e., Days 1–6). “b" indicates *P* < .05 versus ELT of control group for the same day. “c" indicates *P* < .05 versus ELT of 5% Tween 80 group for the same day. Here data were analyzed by ANOVA followed by least significance difference (LSD) test. (c) and (d) *B. monniera* (120 mg kg^−1^ oral) significantly attenuated scopolamine (0.5 and 0.1 mg kg^−1^)-induced impairment of decrease in ELT as compared with respective scopolamine-treated groups. We compared the ELT of *B. monniera-*treated group with scopolamine's ELT in each time point (i.e., Days 1–6). “d" indicates significance of ELT versus same day's ELT of scopolamine (0.5 mg kg^−1^ oral) group. “e" indicates significance of ELT versus same day's ELT of scopolamine (0.1 mg kg^−1^ oral) group. Here data were analyzed by ANOVA followed by LSD *post hoc* test.

**Figure 2:**
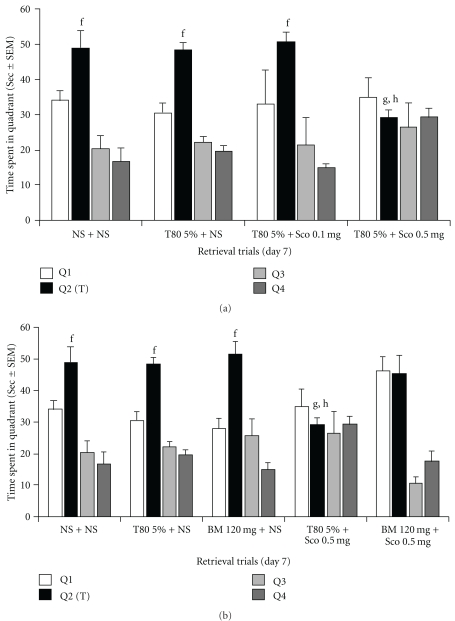
Effect of *B. monniera* on scopolamine-induced retrograde amnesia. During retrieval trials, each value represents mean ± SEM. Control group shows significant enhancement in time spent in target quadrant (Q2) as compared to other quadrant which suggests normal retrieval of memory. The 5% Tween 80 does no alter normal retrieval since this group indicates more time spent in target quadrant that is not different from control group. (a) Scopolamine at dose of 0.5 mg kg^−1^ i.p., but not at 0.1 mg kg^−1^ i.p. dose, significantly reduced the time spent in target quadrant by producing retrograde amnesia. (b) *Bacopa monniera* (120 mg kg^−1^ oral) does not alter normal retrieval as compared to control and 5% Tween 80. *Bacopa monniera* at 120 mg kg^−1^ oral increases the time spent target quadrant in scopolamine treated mice as compared to scopolamine alone treated mice and it, therefore, attenuates scopolamine (0.5 mg kg^−1^ i.p.)-induced retrograde amnesia. “f" indicates *P* < .05 versus time spent in other quadrants, that is, Q1, Q3 and Q4, here data were analyzed using one-way ANOVA followed by Dunnett's test. “g" indicates significance at *P* < .05 versus control group's time spent in target quadrant (Q2). “h" indicates significance at *P* < .05 versus 5% Tween 80 group's time spent in target quadrant (Q2). “i" indicates significance at *P* < .05 versus scopolamine group's time spent in target quadrant (Q2). Here, data were analyzed using one-way ANOVA followed by LSD test.

Control mice showed gradual reduction in ELT with ongoing acquisition trial (“a" indicates *P* < .05 versus ELT on day 1, *F* = 11.26) (Figures 1(a)–1(d)). These mice spent more time in target quadrant as compared to other quadrant (“f" indicates *P* < .05 versus time spent in other quadrant, *F* = 11.48) (Figures 2(a) and 2(b)). It indicates normal acquisition and retrieval on control mice. 5% Tween 80 did not alter gradual decrease in ELT (Figures 1(a)–1(d)) and preferential stay of mice in target quadrant (Figures 2(a) and 2(b)) as compared to control. It suggested the absence of per se effect of 5% Tween 80 on acquisition and retrieval. Similarly, standardized extract of *B. monniera* (120 mg kg^−1^ oral) alone neither enhanced nor impaired the normal acquisition (Figure 1(a)) and retrieval (Figure 2(b)) as compared to control and 5% Tween 80 mice.

The higher doses of scopolamine 1.0 and 0.5 mg kg^−1^ did not show gradual decrease in ELT. Similarly lower doses 0.1 mg kg^−1^ also did not gradually reduce the ELT with acquisition days, although the effect was relatively less intense than the higher doses of scopolamine due to shift in ELT. These groups were significantly different from control and 5% Tween 80 group (b = *P* < .05 and c = *P* < .05, *F* values 1.01, 1.26, 2.89, 3.28, 8.4 and 16.38 on Days 1–6 resp.) (Figure 1(b)). These mice also spent reduced time in target quadrant (Q2) as compared to the control mice when compared on 7th day with other quadrants (Q1, Q3 and Q4) (data not shown). These observations suggest that scopolamine impairs the process of acquisition of new memory by producing anterograde amnesia which subsequently affects retrieval of memory implying lack of acquisition.

For studying the effect of scopolamine on retrieval alone, scopolamine was administered before retrieval trial. Scopolamine at 0.1 mg kg^−1^ (intraperitoneal) i.p. dose did not lower the time spent in target quadrant (Figure 2(a)) where as at 0.5 mg kg^−1^ i.p. it significantly reduced the time spent in target quadrant (Figures 2(a) and 2(b)) when compared to the control group (“g" indicates *P* < .05, *F* = 3.24) and 5% Tween 80 group (“h" indicates *P* < .05, *F* = 3.24).

### 3.2. *Bacopa monniera* Attenuates Scopolamine-Induced Anterograde Amnesia

*Bacopa monniera* at 120 mg kg^−1^ oral (“a” indicates *P* < .001 versus ELT on Day 1, *F* = 16.56) reversed scopolamine (0.5 mg kg^−1^ i.p.)-induced impairment of ELT with ongoing acquisition trials (Figure 1(c)) as compared with scopolamine (0.5 mg kg^−1^ i.p.) group (“d" indicates *P* < .05 versus the same day's ELT of scopolamine group, *F* values were 1.43, 5.38, 4.3, 4.56, 7.29 and 14.5 on Days 1–6 resp.). *Bacopa monniera* at 120 mg kg^−1^ oral also attenuated scopolamine (0.1 mg kg^−1^ i.p.)-induced deficit in decrease in ELT with ongoing acquisition trials (Figure 1(d)) as compared with scopolamine (0.1 mg kg^−1^ i.p.) group (“e" indicates *P* < .05 versus the same day's ELT of scopolamine group, *F* values 1.1, 1.26, 2.89, 3.28, 8.4, 16.38 on Days 1–6 resp.). Interestingly, *B. monniera* showed better antiamnesic effects with 0.1 mg kg^−1^ i.p. dose of scopolamine. These mice also spent more time in target quadrant (Q2) as compared with scopolamine-treated amnesic mice during retrieval trial (data not shown).

### 3.3. *Bacopa monniera* Attenuates Scopolamine-Induced Retrograde Amnesia

*Bacopa monniera* (120 mg kg^−1^ oral) increased the scopolamine affected mice in target quadrant as compared with scopolamine-treated mice (“i" indicates *P* < .05 versus scopolamine group's time spent in target quadrant, *F* = 3.24). *Bacopa monniera* attenuates scopolamine (0.5 mg kg^−1^ i.p.)-induced retrograde amnesia (Figure 2(b)).

## 4. Discussion

Acetylcholine (ACh) is a neurotransmitter that has long received much attention in memory research. Although the effects of ACh on memory have to be regarded separately for the acquisition, consolidation, and recall phase and for different memory systems [38, 39], it remains a fact that ACh acts on cholinergic receptors that are widely distributed throughout in the brain. Cholinergic antagonism is reported to produce cognition deficit which imitates Alzheimer's disease [2] similar to hippocampal lesion-induced cognitive deficits [40, 41]. PSAPP mice, expressing the “Swedish" amyloid precursor protein and M146L presenilin-1 mutations, are a well-characterized model for spontaneous amyloid plaque formation. *Bacopa monniera* extract lowers A–*β* 1–40 and 1–42 levels in cortex and reverses Y-maze performance in PSAPP mice [24]. Similarly it also reduces *β*-amyloid levels in a doubly transgenic mouse model of rapid amyloid deposition (PSAPP mice) by reducing divalent metals, scavenging the reactive oxygen species, decreasing the formation of lipid peroxides and inhibiting lipoxygenase activity [23].

Scopolamine, acetylcholine receptor antagonist, is reported to impair cognitive performances [42–45] especially spatial learning and memory [46, 47]. It exerts amnesic effect equally in various behavioral models of memory including Morris water maze [48–52]. Therefore, scopolamine is considered as reliable tool to study antiamnesic effects of candidate molecules or extracts. Morris water maze model represents the model of memory especially spatial memory. During the acquisition trials, mouse locates the hidden platform using spatial cues. It develops the spatial memory on the basis of spatial arrangement of cues which helps in locating the platform in subsequent trials. In this model, the memory is developed progressively with repetitive trials which resemble the human interactions. This model is very helpful to analyze the reversal amnesic effect with investigational drug because receptive trials with ongoing trials confirm the progress of reversal of amnesia. Moreover, it provides a clean validation platform for comparing the ELT of test and control animals. In order to include the homogenous population of normal mice, we have evaluated the swimming ability of mice in water tank, and muscle coordination in rotating road before performing Morris water maze test. It was selected for Morris water maze test when it showed a good swimming ability and normal amnesic mice, but also impairs the retrieval of trained mice by producing retrograde amnesia. These findings are supported by earlier studies where scopolamine induces cognitive impairment in animal [47, 53] and human [54–56]. It is excluded from the group of study if it did not swim well or rapidly fall from rotating rod due to muscle incoordination. In this study scopolamine significantly impaired the gradual decrease in ELT and subsequently reduced the time spent in target quadrant (Q2) during retrieval trial in water maze tests. These observations suggest that scopolamine not only impairs the process of acquisition by producing anterograde amnesia, which subsequently affects the retrieval of these.

**Figure 3:**
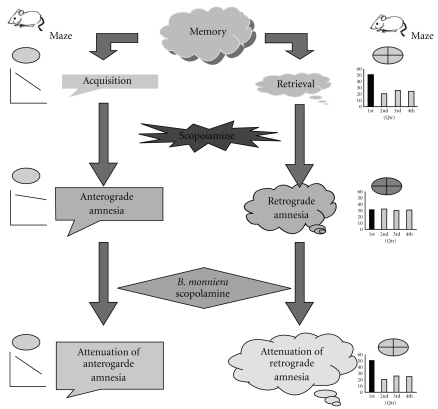
Proposed schematic presentation of effect of *B. monniera* on acquisition and retrieval of memory. The diagram depicts that scopolamine-induced impairment of acquisition and retrieval of memory are reversed by *B. monniera* pretreatment.

*Bacopa monniera* is known to attenuate amnesia [1, 21, 22, 36, 57–59]. In this study we used a suspension of standardized extract powder of *B. monniera*, at 120 mg kg^−1^ oral doses once daily. It is relatively less than non-standardized powder or partial purified products. *Bacopa monniera* shows its effect upon longer treatment rather than short treatment in humans. Therefore, *B. monniera* was administered for 6 days before trials for antiamnesic affect. Our study utilized scopolamine model to evaluate the antiamnesic efficacy of *B. monniera* in consonance with previous approaches [60–64].We report that *B. monniera* able to reverse anterograde and retrograde amnesia induced by scopolamine when administered orally. The proposed scheme of effect of *B. monniera* and scopolamine on acquisition and retrieval of memory is explained in Figure 3. This finding is supported by earlier studies where *B. monniera* extract [21] or its constitutes [65] shows similar effects in animal. *Bacopa monniera* with *ginkgo biloba* extract exerts significant anticholinesterase and antidementic properties in mice and attenuates the scopolamine-induced cognition deficit in passive avoidance test [20] exhibiting cholinergic characteristics [24] besides antioxidant [19, 25–27, 29, 66]. It also attenuates the spatial memory deficit in rat model of Alzheimer's disease [67]. Hosamani and Muralidhara reported the neuroprotective efficacy of *Bacopa monnieri* against rotenone-induced oxidative stress and neurotoxicity in Drosophila melanogaster [68]. *Bacopa monniera* is reported to reverse neurotoxin and colchicines-induced depletion of acetyholine and suppression of choline esterase activity and muscarinic receptor binding in frontal cortex and hippocampus [17, 57]. Moreover, it is documented to inhibit the acetyhcholine esterase activity dose dependently [20]. Endogenous striatal acetylcholine is documented to exert a positive modulatory action on *N*-methyl-d-aspartate (NMDA) responses via muscarinic receptors [69]. *Bacopa monniera* is reported to alter the expression of NR1 subunit of NMDA receptor [70]. *B. monnieri extract* is reported to reverse 5-HT2C receptor mediated motor dysfunction in epilepsy by attenuating 5-HT content, 5-HT2C receptor binding and gene expression in hippocampus of rats [71].

Scopolamine alters the gene expression of various candidate molecules in rat hippocampus, which suggests involvement of cholingergic system in LTP [72]. Several studies strengthens the contribution of cholingergic system for antiamnesic effect of *B. monniera* in accordance with earlier reports [20–22].

We were not able to find any significant effect of *B. monniera* per se on normal acquisition and retrieval of memory. It suggests that *B. monniera* only reverses amnesia but it does not improve the memory of normal animal. It is our assumption that acute treatment could not increase the memory beyond a certain threshold that already exists in a system. Some studies have suggested that the choice of dose and duration of *B. monniera's* treatment is critical for bringing its optimal effects. The high doses of *B. monniera* extract (*∼*50% of LD50) for 15 days demonstrates anticonvulsant activity. When it administer acutely at [73] lower doses (approaching 25% of LD50), anticonvulsant activity is not observed [73]. It is possible that *B. monniera* does not increase the memory after the acute or subchronic treatment, while it alleviates the memory defects. It may enhance the memory after long duration of treatment. *Bacopa monniera* may not increase consolidation sufficient to elicit a significant improvement, although reversal of amnesia may occur by elimination of interference of LTP.

On the basis of our findings, we can conclude that scopolamine-induced amnesia may be mediated by cholinergic system. Although the value of molecular analogs needs more exploration in drug discovery, the potential of traditional knowledge herbs such as *B. monniera* cannot be underscored. It certainly needs deeper investigation, particularly when the current reductionist approach has not resulted in tangible therapies. These may include further studies on downstream signaling mechanisms and other LTP processes.

## Funding

This work was partially supported by the Department of Biotechnology, New Delhi, India (grant no: BT/PR3533/Med/12/152/2002).

## Figures and Tables

**Table 1:** Regimen for administration of vehicle and pharmacological agents.

Treatment	Group	Figure	Administration of vehicle and pharmacological agents	
			Acquisition trials (Days 1–6)	Retrieval trial (Day 7)
Control	I (*n* = 7)	Figures 1A–F, 2(a), and 2(b)	NS (10 mL kg^−1^ PO) 60 min before + NS (10 mL kg^−1^ i.p.) 30 min before	NS (10 mL kg^−1^ PO) 60 min before + NS (10 mL kg^−1^ i.p.) 30 min before
5% Tween 80 per se	II (*n* = 7)	Figures 1A–F, 2(a), and 2(b)	Tween 80 (10 mL kg^−1^ PO) 60 min before + NS (10 mL kg^−1^ i.p.) 30 min before	Tween 80 (10 mL kg^−1^ PO) 60 min before + NS (10 mL kg^−1^ i.p.) 30 min before
Scopolamine anterograde	III-V (*n* = 7/group)	Figures 1(b)–1(d)	Tween 80 (10 mL kg^−1^ PO) 60 min before + Sco (1 mg kg^−1^ i.p., 0.5 mg kg^−1^ i.p., 0.1 mg kg^−1^ i.p.) 30 min before	Tween 80 (10 mL kg^−1^ PO) 60 min before + NS (10 mL kg^−1^ i.p.) 30 min before
Scopolamine retrograde	VI–VII (*n* = 7/group)	Figure 2(b)	Tween 80 (10 mL kg^−1^ PO) 60 min before + NS (10 mL kg^−1^ i.p.) 30 min before	Tween 80 (10 mL kg^−1^ PO) 60 min before + Sco (0.5 mg kg^−1^ i.p., 0.1 mg kg^−1^ i.p.) 30 min before
*Bacopa monniera* + Scopolamine 0.5 mganterograde	VIII (*n* = 7)	Figure 2(b)	bm (120 mg kg^−1^ PO) 60 min before + Sco (0.5 mg kg^−1^ i.p.) 30 min before	Tween 80 (10 mL kg^−1^ PO) 60 min before + NS (10 mL kg^−1^ i.p.) 30 min before
*Bacopa monniera* + Scopolamine 0.1 mganterograde	IX (*n* = 7)	Figure 2(b)	BM (120 mg kg^−1^ PO) 60 min before + Sco (0.1 mg kg^−1^ i.p.) 30 min before	Tween 80 (10 mL kg^−1^ PO) 60 min before + NS (10 mL kg^−1^ i.p.) 30 min before
*Bacopa monniera* + Scopolamine 0.5 mgretrogarde	X (*n* = 7)	Figures 2E and F	Tween 80 (10 mL kg^−1^ PO) 60 min before + ns (10 mL kg^−1^ i.p.) 30 min before	BM (120 mg kg^−1^ PO) 60 min before + Sco (0.5 mg kg^−1^ i.p.) 30 min before
*Bacopa monniera*−per se	XI (*n* = 7)	Figures 1(a), 2(b)	BM (80 mg, 120 mg kg^−1^ PO) 60 min before	BM (80 mg, 120 mg kg^−1^ PO) 60 min before

BM: *Bacopa monniera* standardized extract suspension in 5% Tween 80, NS: normal saline, *n*: number of mouse; Sco: scopolamine solution in normal saline; Tween 80: 5% v/v Tween solution in normal saline.
